# Impaired Kidney Function, Subclinical Myocardial Injury, and Their Joint Associations with Cardiovascular Mortality in the General Population †

**DOI:** 10.3390/jcm14197123

**Published:** 2025-10-09

**Authors:** Ahmed E. Shatta, Mohamed A. Mostafa, Mohamed A. Attia, Tarek Ahmad Zaho, Richard Kazibwe, Elsayed Z. Soliman

**Affiliations:** 1 Epidemiological Cardiology Research Center, Department of Cardiovascular Medicine, Wake Forest School of Medicine, Winston Salem, NC 27157, USA; ahmed.shata@live.com (A.E.S.); mmostafa@wakehealth.edu (M.A.M.); mohamedashraf511@gmail.com (M.A.A.); tzaho@wakehealth.edu (T.A.Z.); 2 Section on Hospital Medicine, Department of Medicine, Wake Forest School of Medicine, Winston Salem, NC 27157, USA; richard.kazibwe@advocatehealth.org

**Keywords:** subclinical myocardial injury, kidney function, cardiovascular mortality, NHANES-III

## Abstract

**Background:** The combined impact of impaired kidney function and subclinical myocardial injury (SCMI) on cardiovascular (CV) mortality has not been well studied. We aimed to evaluate their individual and joint associations with cardiovascular mortality. **Methods:** We analyzed data from 6057 participants (mean age 57.0 ± 13.0 years) in the U.S. Third National Health and Nutrition Examination Survey. Estimated glomerular filtration rate (eGFR) was calculated using the CKD-EPI equation. Electrocardiographic SCMI was defined as a cardiac infarction/injury score ≥ 10. CV mortality was determined from the National Death Index. Multivariable logistic regression assessed baseline cross-sectional associations between eGFR and SCMI. Cox proportional hazards models were used to examine the individual and combined associations of eGFR and SCMI with CV mortality. **Results:** At baseline, 1297 participants (21.4%) had SCMI. In multivariable logistic regression analysis, eGFR < 45 mL/min/1.73 m^2^ (vs. ≥45) was not associated with SCMI (OR [95% CI]: 1.10 [0.84–1.45]). Over a median follow-up of 18.4 years, 690 CV deaths occurred. In separate Cox models, both SCMI (vs. no SCMI) and eGFR < 45 (vs. ≥45) were associated with increased CV mortality risk (HR [95% CI]: 1.36 [1.16–1.60] and 1.56 [1.24–1.99], respectively). Compared with participants with eGFR ≥ 45 and no SCMI, those with both eGFR < 45 and SCMI had the highest CV mortality risk (HR [95% CI]: 2.36 [1.65–3.36]), followed by eGFR < 45 alone (1.47 [1.09–1.96]) and SCMI alone (1.33 [1.11–1.58]). **Conclusions:** Both reduced eGFR and SCMI were independently associated with CV mortality. Their coexistence showed the highest risk, but without statistical significance compared with each alone, possibly reflecting limited power and distinct mechanisms.

## 1. Introduction

Impaired kidney function has been consistently linked to an elevated long-term risk of cardiovascular disease (CVD), with cardiovascular mortality being the leading cause of death among individuals with kidney disease [1]. Cardiovascular complications, particularly coronary artery disease (CAD), are among the most prevalent outcomes observed in this population [2,3]. The mechanisms underlying this association are multifactorial and may involve both direct effects such as volume overload, hypertension, vascular calcification, and accelerated atherosclerosis that contribute to structural and functional cardiac deterioration, as well as indirect effects through the exacerbation of traditional cardiovascular risk factors [4,5,6,7,8].

Recent studies further demonstrate that chronic kidney disease (CKD) is consistently associated with an increased risk of sudden cardiac arrest (SCA), underscoring the arrhythmogenic burden of kidney disease [9]. This highlights that beyond atherosclerotic complications such as CAD, CKD is also strongly linked to arrhythmic and subclinical myocardial injury pathways, reinforcing the need to better understand early cardiac damage in this population.

While overt cardiovascular events have been extensively studied in patients with CKD, less is known about the early and often silent manifestations of cardiac injury. Subclinical myocardial injury (SCMI), characterized by subclinical ischemic myocardial damage in the absence of overt symptoms, is a significant predictor of adverse cardiovascular outcomes, including CVD mortality [10,11]. SCMI can be identified using the Cardiac Infarction/Injury Score (CIIS), a validated electrocardiographic marker of myocardial injury that has been widely applied in epidemiological studies [11,12].

In parallel, circulating biomarkers such as cardiac troponin serve as indicators of subclinical cardiovascular injury. Elevated resting troponin concentrations are particularly common in patients with CKD and have been linked to measures of subclinical cardiac dysfunction, highlighting the presence of silent myocardial damage in this population [13].

Whether impaired kidney function is associated with SCMI is currently unknown, and the combined impact of both conditions on CV mortality has not been well studied. We addressed these questions using data from the United States Third National Health and Nutrition Examination Survey (NHANES III). The purpose of this study was to assess the individual and combined associations of impaired kidney function and SCMI with CV mortality in the general population. In addition, we aimed to determine whether SCMI provides incremental value for cardiovascular risk stratification, particularly in individuals with reduced kidney function, thereby informing the potential clinical utility of incorporating ECG-based measures into CKD care pathways.

## 2. Methods

### 2.1. Study Population

NHANES III is a program designed to assess health and nutritional status among U.S. adults and children living in the community. It is conducted by the National Center for Health Statistics (NCHS), part of the Centers for Disease Control and Prevention. NHANES III was carried out between 1988 and 1994. All participants gave written informed consent, and the study protocol was approved by the National Center for Health Statistics (NCHS) institutional review board. Further information on the survey’s design, methodology, and data availability has been documented in prior publications [14,15].

For the purpose of this analysis, we included NHANES III participants with available ECG data at baseline. By design, only NHANES-III participants aged 40 years or older underwent ECG recording. We excluded those with prior CVD (prior myocardial infarction, heart failure or stroke) and those with missing key variables needed for analysis, those with eGFR < 15 mL/min/1.73 m^2^ and those with major ECG abnormalities. After all exclusions, 6057 participants were included in the analysis.

### 2.2. Ascertainment of Kidney Function

During NHANES-III assessments either in house or mobile units, blood samples were collected via venipuncture by phlebotomist and samples were analyzed for biomarkers including creatinine level. Renal function was assessed using eGFR, which was calculated from serum creatinine using the Chronic Kidney Disease Epidemiology Collaboration (CKD-EPI) equation [16]. Participants were stratified into two groups: eGFR ≥ 45 mL/min/1.73 m^2^ and eGFR < 45 mL/min/1.73 m^2^.

### 2.3. Defining Subclinical Myocardial Injury (SCMI)

Electrocardiographic Cardiac Infarction/Injury Score (CIIS) score was used to define SCMI [10,11,12]. In NHANES-III, a resting 12-lead ECG was obtained using a Marquette MAC 12 electrocardiograph (Marquette Medical Systems, Milwaukee, WI, USA) during a physical examination conducted within a mobile examination center. The digital signals of the ECG tracings were sent to the Epidemiological Cardiology Research Center (EPICARE) of Wake Forest University School of Medicine (Winston-Salem, NC, USA) for central processing. ECGs underwent visual inspection by skilled technicians before being automatically processed using GE 12-SL program (Marquette Medical Systems, Milwaukee, WI, USA).

The CIIS is a weighted scoring system designed to assess the likelihood of myocardial injury or ischemia based on quantitative ECG features. It incorporates a total of 15 components, including 11 discrete and 4 continuous ECG variables, derived from standard 12-lead ECGs. These elements capture abnormalities in Q waves, R waves, T waves, and the ST segment, and together provide a risk-stratified assessment of cardiac injury. The CIIS is structured to allow for both manual interpretation and automated processing, making it suitable for use in large population-based studies [12].

In NHANES III dataset, CIIS values were initially stored after being multiplied by 10 to avoid the use of decimal points in the database. For the purpose of this analysis, the values were converted back to their original scale by dividing by 10 [5]. In accordance with established definitions in the literature, a CIIS of 10 or greater was used to define the presence of SCMI) [5,11,17,18].

### 2.4. Ascertainment of Cardiovascular Mortality

The primary outcome was CV mortality, determined through linkage to the National Death Index with follow-up through 31 December 2015. CV deaths were identified using ICD-9 and ICD-10 codes corresponding to underlying causes of death.

### 2.5. Other Variables

Demographics (age, sex, race), and smoking status, education level, family income and medication intake were self-reported during an in-home interview. Physical activity was assessed based on the frequency of leisure time activity and included information on types of activity, frequency, and level of activity. Body mass index (BMI) was defined as height over square root of weight using data from a physical examination conducted at a mobile examination center. Systolic and diastolic blood pressure were measured while seated, and up to three measurements were averaged. Diabetes mellites was defined as fasting blood glucose levels ≥126 mg/dl or the use of glucose-lowering medications. Total cholesterol, creatinine and glucose, and other components in the metabolic panel using laboratory procedures as reported by the National Center for Health Statistics [15].

### 2.6. Statistical Analysis

We compared participant characteristics stratified by the presence of SCMI using Chi-square tests for categorical variables. For continuous variables, we first assessed distributional assumptions using Q–Q plots and formal tests of normality. We summarized the variables that were not normally distributed as medians with interquartile ranges (IQRs) and compared groups using the Wilcoxon rank-sum test. Categorical variables are presented as counts and percentages.

Multivariable logistic regression was used to assess the cross-sectional association between levels of eGFR and the presence SCMI. We modeled eGFR as both categorical (eGFR ≥ 45 vs. <45) mL/min/1.73 m^2^ and as a continuous variable (per 1 SD decrease). To provide additional stratification and clinical context, we also performed sensitivity analyses using eGFR quartiles. These results are presented in Supplementary Table S1 and Supplementary Figure S1. Models were adjusted as follows: Model 1 was adjusted for sociodemographic variables: age, sex, race/ethnicity, and education. Model 2 was further adjusted for traditional cardiovascular risk factors, including diabetes, systolic blood pressure, antihypertensive medication use, BMI, lipid-lowering medication use, smoking status, and physical activity.

We calculated CV mortality incidence rates per 1000-person years stratified by eGFR levels and SCMI status. Multivariable Cox-proportional hazard models were used to assess the associations of low levels of eGFR and SCMI, separately and in combination with CV mortality. Model 1 adjusted for sociodemographic variables: age, sex, race/ethnicity, and education. Model 2 was further adjusted for traditional cardiovascular risk factors, including diabetes, systolic blood pressure, antihypertensive medication use, BMI, lipid-lowering medication use, smoking status, and physical activity.

To evaluate the clinical utility of SCMI, we compared two logistic regression models for predicting cardiovascular death. The baseline model included our Model 2 variables (age, sex, race, education, diabetes, antihypertensive use, systolic blood pressure, body mass index, lipid-lowering therapy, smoking, total cholesterol, and physical activity). The extended model added SCMI to these predictors. We assessed discrimination using the C-statistics and quantified changes in risk classification with the continuous net reclassification improvement (NRI).

All statistical analyses were performed using SAS version 9.4 (SAS Institute Inc., Cary, NC, USA). A two-sided α of 0.05 was used for hypothesis testing.

## 3. Results

This analysis included 6057 participants (mean age 57.0 ± 13.0 years); 54.4% women, 50% non-Hispanic whites). A total of 1297 (21.4%) individuals had prevalent SCMI at baseline. Participants with SCMI tended to be older, with higher prevalence diabetes, and higher levels of systolic blood pressure, total cholesterol and BMI (Table 1).

In logistic regression models adjusted for sociodemographic and CV risk factors, low eGFR (<45 mL/min/1.73 m^2^) was not associated with increased risk of SCMI compared to those with eGFR ≥ 45 mL/min/1.73 m^2^. Similar results were obtained when eGFR was used in the model per 1-SD decrease (Table 2).

After a median follow-up of 18.2 years, 690 (11.4%) participants experienced CV mortality. In Cox proportional hazards models, participants with SCMI had a 43% higher risk of CV death compared to those without SCMI, a finding that remained significant in fully adjusted models (Table 3). Similarly, and in a separate analysis, participants with low eGFR (eGFR < 45 mL/min/1.73 m^2^) had a higher CV mortality rate compared to those with eGFR ≥ 45 mL/min/1.73 m^2^ (32% vs. 10%) with an increased risk of CV mortality by 56%) in fully adjusted models accounting for sociodemographic and CV risk factors, (Table 4). Modeling eGFR as a continuous variable, each 1-SD decrease in eGFR was associated with a 14% higher risk of CV mortality in a linear pattern, supporting a dose–response relationship.

**Figure 1:**
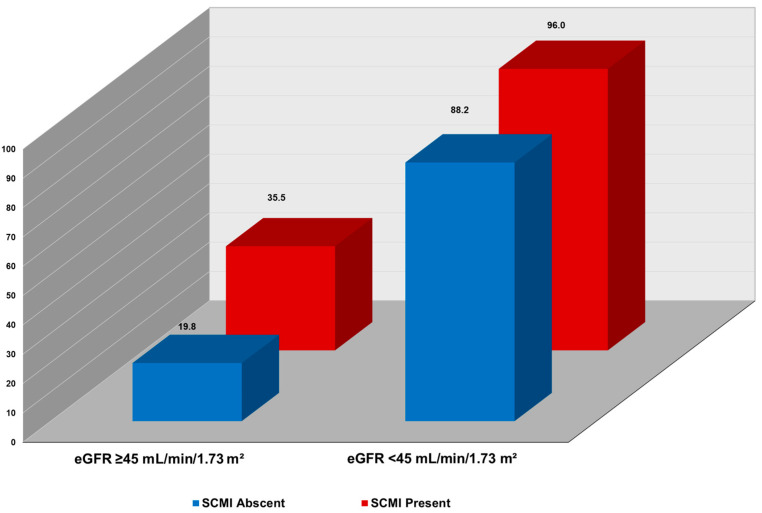
Cardiovascular Mortality Rates Stratified by eGFR Levels and SCMI Status. Incidence rate per 1000 person-years; SCMI = subclinical myocardial injury, eGFR = estimate glomerular filtration rate.

In joint analysis, incremental, stepwise increase in CV mortality was observed across combinations of SCMI and eGFR levels. As shown in Figure 1, the highest CV mortality rate per 1000-person-year occurred in participants with both eGFR < 45 mL/min/1.73 m^2^ and SCMI. In contrast, the lowest CV mortality rate was in those with eGFR ≥ 45 mL/min/1.73 m^2^ and without SCMI. Also, in multivariable Cox proportional hazard analysis, participants with both SCMI and eGFR < 45 mL/min/1.73 m^2^ had more than double the risk of CV death (HR 2.36; 95% CI, 1.65–3.36; *p* < 0.001) than those without SCMI and with eGFR ≥ 45 mL/min/1.73 m^2^. Other combinations of eGFR < 45 mL/min/1.73 m^2^ and presence of SCMI status were also associated with increased risk of CV mortality compared eGFR eGFR ≥ 45 mL/min/1.73 m^2^ and absence of SCMI (Table 5). The coexistence of both conditions was associated with the highest observed risk; however, this difference was not statistically significant compared with either condition alone. In supplementary analyses stratifying eGFR into quartiles, results were consistent with the primary findings, showing higher cardiovascular mortality risk in lower quartiles and the highest observed risk in participants with both reduced eGFR and SCMI (Supplementary Table S1, Supplementary Figure S1).

In evaluating model performance, the baseline model showed good discrimination (C-statistic 0.79), and adding SCMI led to only a negligible change (ΔC = +0.002). Reclassification analysis, however, suggested a modest benefit: fewer events were correctly reclassified (NRI_events = −0.37), but more nonevents were correctly reclassified downward (NRI nonevents = +0.60), yielding an overall NRI of +0.23.

## 4. Discussion

CV mortality remains the primary cause of death globally and accounts for excess mortality among individuals with impaired kidney function. In this nationally representative analysis, both reduced eGFR and the presence of SCMI were independently associated with increased risk of CV mortality. Importantly, when both conditions coexisted, the risk of CV death was highest. However, the difference compared with either condition alone did not reach statistical significance, which may reflect limited power to detect incremental risk. Despite their shared impact on CV mortality, baseline lower levels of eGFR were not associated with the presence of SCMI. The lack of association between eGFR and SCMI suggests that their combined contribution to the risk of CVD may operate through different pathways.

While the relationship between poor kidney function and adverse cardiovascular outcomes is well established and generally progresses linearly with CKD stage, our findings showed no significant association between lower levels of eGFR and increased prevalence of SCMI. It is important to note that the prior literature has predominantly focused on overt CVD outcomes [1,2,3,4,5], while subclinical disease processes such as SCMI is mainly underexplored. Differences in the associations of CKD with overt CVD versus SCMI probably reflect differences in pathophysiological pathways and disease timelines. While reduced eGFR contributes to cardiac remodeling through mechanisms such as volume overload, arterial stiffness, mineral dysregulation, and systemic inflammation, SCMI likely results from focal ischemic injury or microvascular compromise, processes that may not directly correlate with kidney disease biomarkers [19]. Additionally, individuals with low eGFR are often targeted for intensive cardiovascular monitoring and early preventive therapy, which may attenuate or delay the development of detectable SCMI, potentially masking any direct relationship [20].

Some reports have shown that subclinical cardiovascular disease, defined as elevated cardiac biomarkers, was associated with a significantly faster rate of kidney function decline. This raises the possibility of bidirectional or reversed causality, where SCMI contributes to renal dysfunction, rather than the other way around, and provides evidence that structural and functional cardiac changes may adversely affect kidney function [21,22]. Such complexity reinforces the need to better understand the temporal and mechanistic links between subclinical cardiac diseases and kidney function.

Our finding of a significant association between reduced eGFR and CV mortality, together with supplementary analyses using eGFR quartiles that provided additional granularity and confirmed consistency with the primary results, aligns with prior studies demonstrating the prognostic importance of kidney dysfunction in CV outcomes [23,24,25]. In a retrospective study by Huang et al., eGFR < 45 mL/min/1.73 m^2^ had approximately a 70% higher risk of CV mortality compared to individuals with preserved kidney function [25]. Also, the Framingham Heart Study has demonstrated nearly 40–110% increase in CV mortality even in those with mildly reduced eGFR (60–89 mL/min/1.73 m^2^) [26]. These findings emphasize the broad predictive value of renal function markers across clinical and subclinical stages [1,2,3,4,5,6,7,8]. The possible mechanisms by which lower eGFR contributes to CV risk include arterial stiffness, widened pulse pressure, impaired coronary perfusion, and left ventricular hypertrophy [23,24]. These changes promote myocardial strain, ischemia, and arrhythmogenic remodeling, all of which elevate long-term risk for cardiovascular events.

Our observed association of SCMI with increased CV mortality was also consistent with the previous literature [8,12,17]. SCMI reflects a state of chronic, subclinical myocardial injury, often driven by microvascular dysfunction, myocardial fibrosis, or autonomic imbalance. The pathophysiological basis linking SCMI to CV mortality may involve several interrelated mechanisms including the cumulative myocardial damage due to repeated silent ischemia or underlying structural abnormalities such as fibrosis and ventricular remodeling [27]. These alterations can impair myocardial function, reduce contractile reserve, and promote electrical instability, increasing the risk for worsening CVD and mortality [28,29]. SCMI may also reflect systemic atherosclerotic burden and autonomic dysregulation, both of which are independently associated with adverse CV outcomes [30,31].

Of note, participants with SCMI showed higher systolic blood pressure at baseline, which may partly reflect their lower use of antihypertensive therapy. Although our models adjusted for blood pressure medication use, it remains possible that the type and intensity of cardiovascular medications could modify the association between SCMI and outcomes. Certain agents, such as beta-blockers, RAAS inhibitors, and statins, may exert protective effects through mechanisms beyond blood pressure lowering, including improvements in vascular remodeling, autonomic regulation, and renal–cardiac cross talk [32,33]. Future studies are warranted to evaluate whether specific therapies attenuate the excess cardiovascular risk observed with concomitant SCMI and reduced kidney function.

While both eGFR and SCMI were independently linked to mortality, a notable finding in our analysis was that individuals with both reduced eGFR and concurrent SCMI had the highest observed risk of CV mortality compared to those with neither condition. However, the difference compared with either condition alone was not statistically significant, which may be due to limited statistical power. This highest observed risk may reflect overlapping mechanisms such as systemic inflammation, endothelial dysfunction, and hemodynamic stress, as well as distinct pathological pathways specific to each condition [1,2,3,4,5,6,7,8,25,26,27,28,29]. SCMI may contribute additional ischemic burden and structural remodeling beyond that already imposed by kidney disease. At the same time, our performance metrics suggest that the primary value of SCMI may lie less in improving overall discrimination and more in refining risk classification, particularly through better identification of individuals unlikely to experience cardiovascular death. This pattern highlights a potential role for SCMI in reducing overtreatment by strengthening risk stratification at the lower end of the spectrum. This observation parallels reports where CKD combined with other subclinical markers, such as coronary artery calcification or elevated troponin, resulted in disproportionate increases in risk, reinforcing the concept that overlapping subclinical abnormalities magnify cardiovascular risk [34].

Taken together, our findings emphasize the value of SCMI as a clinically meaningful marker of cardiovascular vulnerability in patients with impaired kidney function. Incorporating SCMI assessment using ECG-based scores may enhance cardiovascular risk stratification in this population. Given its low cost, broad availability, and non-invasive nature, the electrocardiogram presents a practical method for identifying SCMI in individuals without overt heart disease. Although the CIIS is a validated and cost-effective method for identifying SCMI, its complexity may limit application in routine clinical settings. Future research should focus on developing simplified or automated tools, potentially through AI-based ECG analysis and machine learning approaches that could make SCMI detection more accessible and clinically practical. In the context of escalating healthcare costs, screening for subclinical cardiovascular disease markers such as SCMI may offer a cost-effective approach for detecting high-risk individuals.

From a translational perspective, SCMI detection could be integrated into CKD care pathways, especially among high-risk subgroups such as those with eGFR below 45 mL/min/1.73 m^2^. In this group, the coexistence of reduced eGFR and SCMI was associated with nearly double the risk of cardiovascular mortality, emphasizing the importance of confirmatory evaluation (for example, repeat ECG or appropriate cardiac imaging) and intensification of proven preventive therapies, including statin initiation and strict blood pressure control. At the same time, our performance metrics suggest that SCMI may also be clinically valuable at the lower end of the risk spectrum by helping identify individuals unlikely to experience cardiovascular death. In this context, SCMI could reduce overtreatment and allow for more efficient allocation of preventive resources. This dual role—guiding intensification of therapy in high-risk patients while minimizing unnecessary interventions in low-risk individuals—underscores the potential for SCMI screening to refine risk stratification in CKD care. This approach could provide a framework for testing whether SCMI-guided strategies ultimately reduce cardiovascular events. Future research may explore how SCMI screening could be incorporated into existing CKD management frameworks, and whether such an approach might support more tailored preventive strategies, improve resource allocation, and contribute to reductions in cardiovascular risk.

Although our study was not designed to directly compare the incremental predictive value of SCMI against established risk tools such as ASCVD or CKD staging, our results highlight its potential as a complementary marker. By identifying high-risk individuals who may otherwise be underestimated by conventional scores, SCMI could serve to refine cardiovascular risk stratification, especially in the setting of impaired kidney function.

## 5. Limitations

This study should be interpreted in the context of several limitations. First, kidney function was assessed using a single eGFR measurement, which may not fully capture long-term renal function or reflect dynamic changes over time. Incorporating imaging modalities or additional biomarkers such as albuminuria or cystatin C could provide a more comprehensive assessment of kidney health.

Second, SCMI was determined based on a single ECG, which introduces potential intra-individual variability and raises concerns about reproducibility and inter-rater reliability. Such variability may stem from technical factors (e.g., minor differences in electrode placement) or biological fluctuations such as respiration or positional changes. To minimize these sources of error, all ECGs were obtained under standardized acquisition protocols and analyzed at the EPICARE Reading Center, where rigorous quality control procedures were applied, including automated scoring, expert manual review, and exclusion of poor-quality tracings. SCMI was defined using the CIIS and the Minnesota Code, both of which are well-validated systems that have demonstrated good-to-excellent reproducibility and prognostic relevance. The absence of complementary biochemical markers such as cardiac troponins remains a limitation; however, the CIIS has been validated against clinical outcomes and provides a robust measure of subclinical myocardial injury in large-scale epidemiological studies.

Additionally, our analysis did not explicitly model competing risks from non-cardiovascular death. Although cause-specific Cox regression is a standard approach for evaluating cardiovascular mortality, it assumes independence between competing causes of death. Given the long follow-up and older age of many participants, non-cardiovascular mortality could have influenced the observed associations.

Lastly, although the models were adjusted for a wide range of sociodemographic and cardiovascular risk factors, residual confounding due to unmeasured variables such as albuminuria, systemic inflammatory biomarkers, and medication exposures, i.e., RAAS inhibitors or statins could have influenced the observed associations.

Despite these limitations, the key strengths of the study include the use of a large, diverse, nationally representative sample and the application of validated methods for outcome and exposure assessment.

## 6. Conclusions

In this analysis of NHANES III participants, both reduced eGFR and the presence of SCMI were independently associated with increased risk of cardiovascular mortality. When both conditions coexisted, the risk of CV death was highest. These findings highlight SCMI as a potentially valuable marker for identifying individuals at a higher cardiovascular risk, particularly in the setting of impaired kidney function. Incorporating SCMI into routine risk assessment may enhance the early identification of high-risk patients and support more targeted prevention strategies.

## Figures and Tables

**Table 1:** Baseline characteristics of the study participants.

Characteristics	SCMI Absent (*n* = 4760)	SCMI Present (*n* = 1297)	*p*-Value
Age in years (IQR)	56.0 (46.0–67.0)	56.0 (46.0–67.0)	<0.001
Women (%)	2636 (55.4%)	660 (50.9%)	0.004
Race Ethnicity:			
Non-Hispanic White (%)	2320 (48.7%)	709 (54.7%)	<0.001
Non-Hispanic Black (%)	1009 (21.2%)	289 (22.3%)	
Mexican American (%)	1200 (25.2%)	271 (20.9%)	
Other (%)	231 (4.9%)	28 (2.2%)	
Ever Smoked (%)	2257 (47.4%)	523 (40.3%)	<0.001
Body Mass Index (kg/m^2^)	26.8 (23.9–30.3)	26.8 (23.9–30.3)	0.1160
Systolic Blood Pressure (mm Hg)	127.0 (117.0–141.0)	127.0 (117.0–141.0)	<0.001
Diastolic Blood Pressure (mm Hg)	76.0 (70.0–82.0)	76.0 (70.0–82.0)	0.8129
Total Cholesterol (mg/dL)	220.0 (192.0–247.0)	220.0 (192.0–247.0)	0.0341
Diabetes Mellitus (%)	609 (12.8%)	232 (17.9%)	<0.001
Use of BP Medications (%)	3892 (81.8%)	956 (73.7%)	<0.001
Use of lipid lowering (%)	194 (4.1%)	46 (3.6%)	0.387
eGFR < 45 mL/min/1.73 m^2^ (%)	197 (4.1%)	93 (7.2%)	<0.001

SCMI: subclinical myocardial infarction. eGFR: estimated glomerular filtration rate. BP: blood pressure. Categorical variables presented as numbers and percentage while continuous variables presented as median (interquartile range).

**Table 2:** Association of eGFR levels with SCMI.

eGFR Level	SCMI N (%)		Model 1		Model 2	
	Present	Absent	OR (95% CI)	*p*-Value	OR (95% CI)	*p*-Value
eGFR ≥ 45 mL/min/1.73 m^2^	1204 (20.9%)	4563 (79.1%)	Ref.	--	Ref.	--
eGFR < 45 mL/min/1.73 m^2^	93 (7.2%)	197 (4.1%)	1.17 (0.89–1.53)	0.267	1.10 (0.84–1.45)	0.482
eGFR per 1-SD decrease *	--		1.03 (0.95–1.11)	0.447	1.02 (0.94–1.10)	0.664

SCMI = subclinical myocardial injury. eGFR = estimate glomerular filtration. OR = Odds Ratio, CI = Confidence Interval. Model 1adjusted for age, sex, race (white vs. nonwhite), and educational attainment (high school education). Model 2 was additionally adjusted diabetes status, use of antihypertensive medications, systolic blood pressure, body mass index, use of lipid-lowering medications, smoking status, total cholesterol, and physical activity. * 1-SD eGFR = 15.6 mL/min/1.73 m^2^.

**Table 3:** Association Between SCMI and Cardiovascular Mortality.

Outcome	Events N (%)	Model 1		Model 2	
		HR (95% CI)	*p*-Value	HR (95% CI)	*p*-Value
SCMI-Absent	472 (9.9)	Reference	--	Reference	--
SCMI-Present	218 (16.8)	1.43 (1.23–1.68)	<0.001	1.36 (1.16–1.60)	<0.001

SCMI = subclinical myocardial injury. HR = Hazard Ratio, CI = Confidence Interval. Model 1 adjusted for age, sex, race (white vs. nonwhite), and educational attainment (high school education). Model 2 additionally adjusted diabetes status, use of antihypertensive medications, systolic blood pressure, body mass index, use of lipid-lowering medications, smoking status, total cholesterol, and physical activity.

**Table 4:** Association Between eGFR and Cardiovascular Mortality.

eGFR Level	Events N (%)	Model 1		Model 2	
		OR (95% CI)	*p*-Value	OR (95% CI)	*p*-Value
eGFR ≥ 45 mL/min/1.73 m^2^	596 (10.3%)	Reference	--	Reference	--
eGFR < 45 mL/min/1.73 m^2^	94 (32.4%)	1.61 (1.27–2.03)	<0.001	1.56 (1.24–1.99)	<0.001
eGFR per 1-SD decrease *		1.14 (1.03–1.55)	0.013	1.14 (1.03–1.25)	0.010

eGFR = estimate glomerular filtration rate, OR = Odds Ratio, CI = Confidence Interval. Model 1 adjusted for age, sex, race (white vs. nonwhite), and educational attainment (high school education). Model 2 additionally adjusted diabetes status, use of antihypertensive medications, systolic blood pressure, body mass index, use of lipid-lowering medications, smoking status, total cholesterol, and physical activity. * 1-SD eGFR = 15.6 mL/min/1.73 m^2^.

**Table 5:** Association of Combinations of eGFR Levels and SCMI Status with Cardiovascular Mortality.

eGFR and SCMI Status	Participants (n)/Events (%)	Model 1		Model 2	
		HR (95% CI)	*p*-Value	HR (95% CI)	*p*-Value
SCMI Absent + eGFR ≥ 45	4563/414 (9.1%)	Reference	--	Reference	--
SCMI Present + eGFR ≥ 45	1204/182 (15.1%)	1.40 (1.18–1.66)	<0.001	1.33 (1.11–1.58)	0.002
SCMI Absent + eGFR < 45	197/58 (29.4%)	1.55 (1.16–2.07)	0.0031	1.47 (1.09–1.96)	0.011
SCMI Present + eGFR < 45	93/36 (38.7%)	2.40 (1.69–3.41)	<0.001	2.36 (1.65–3.36)	<0.001

SCMI = subclinical myocardial injury, eGFR = estimate glomerular filtration rate, HR = Hazard Ratio, CI = Confidence Interval. Model 1 adjusted for age, sex, race (white vs. nonwhite), and educational attainment (high school education). Model 2 additionally adjusted diabetes status, use of antihypertensive medications, systolic blood pressure, body mass index, use of lipid-lowering medications, smoking status, total cholesterol, and physical activity.

## Data Availability

Data used in this study are publicly available at https://wwwn.cdc.gov/nchs/nhanes/nhanes3/datafiles.aspx, accessed on 1 March 2023.

## Supplementary Materials

The following supporting information can be downloaded at https://www.mdpi.com/article/10.3390/jcm14197123/s1, Figure S1: CV mortality incidence rates stratified by eGFR quartiles and SCMI status. Figure S1 footnote: Incidence rate per 1000 person-years. Table S1: Association of combinations of eGFR quartiles and SCMI Status with CVD mortality.
