# Supplementary material for: Impaired Kidney Function, Subclinical Myocardial Injury, and Their Joint Associations with Cardiovascular Mortality in the General Population
Source: J Clin Med. 2025 Oct 9;14(19):7123. doi: 10.3390/jcm14197123 (PMC12525244; doi:10.3390/jcm14197123)
Supplement: Supplementary file 1 [file jcm-14-07123-s001.zip › jcm-3866349-supplementary.pdf]

Associations of Renal Function with Subclinical Myocardial Injury and their Joint Associations with Cardiovascular Mortality

Supplementary materials:

1- Supplementary Table S1: Association of combinations of eGFR quartiles and SCMI Status with CVD mortality.
2- Supplementary Figure S1. CV mortality incidence rates stratified by eGFR quartiles and SCMI status.

Supplementary Table S1: Association of combinations of eGFR quartiles and SCMI Status with CVD mortality

AIP and SCMI Status	Participants (n)/ Events (%)	Model 1		Model 2	
		HR (95%CI)	*P-value*	HR (95%CI)	*P-value*
SCMI Absent + eGFR-Q4	1492/68(4.6)	Reference	--	Reference	--
SCMI Absent + eGFR-Q3	1481/110(7.4)	1.05(0.77 – 1.42)	0.885	1.06 (0.78 – 1.44)	0.702
SCMI Absent + eGFR-Q2	1374/161(11.7)	0.98(0.73 – 1.32)	0.766	1.02 (0.75 – 1.37)	0.920
SCMI Absent + eGFR-Q1	1215/257(21.2)	1.40 (1.03– 1.88)	0.029	1.32(0.97 – 1.78)	0.078
SCMI Present + eGFR-Q4	388/42(10.8)	1.63(1.11 – 2.40)	0.014	1.53(1.04 – 2.26)	0.031
SCMI Present + eGFR-Q3	419/58(13.8)	1.61(1.13 – 2.30)	0.009	1.54(1.08 – 2.21)	0.018
SCMI Present+ eGFR-Q2	503/96(19.1)	1.55 (1.11 – 2.15)	0.009	1.47(1.05-2.04)	0.023
SCMI Present+ eGFR-Q1	674/188(27.9)	1.88(1.38 – 2.57)	<0.001	1.70(1.24-2.33)	0.001

SCMI = subclinical myocardial injury, AIP = Atherogenic Index of Plasma, CVD = cardiovascular disease, HR = Hazard Ratio, CI = Confidence Interval

eGFR Q1 (<57.5 mL/min/1.73 m²] (n=1889)
eGFR Q2 (57.5 to 63.4 mL/min/1.73 m²] (n=1877)
eGFR Q3 (63.5 to 79.8 mL/min/1.73 m²] (n=1900)
eGFR Q4 (≥79.9 mL/min/1.73 m²] (n=1880)

***Model 1** adjusted for age, sex, race and education level. **Model 2** adjusted for model 1 plus history of diabetes, systolic blood pressure, use of antihypertension medication, body mass index, use of lipid lowering medications, smoking status, and physical activity.*

Supplementary Figure S1. CV mortality incidence rates stratified by eGFR quartiles and SCMI status.

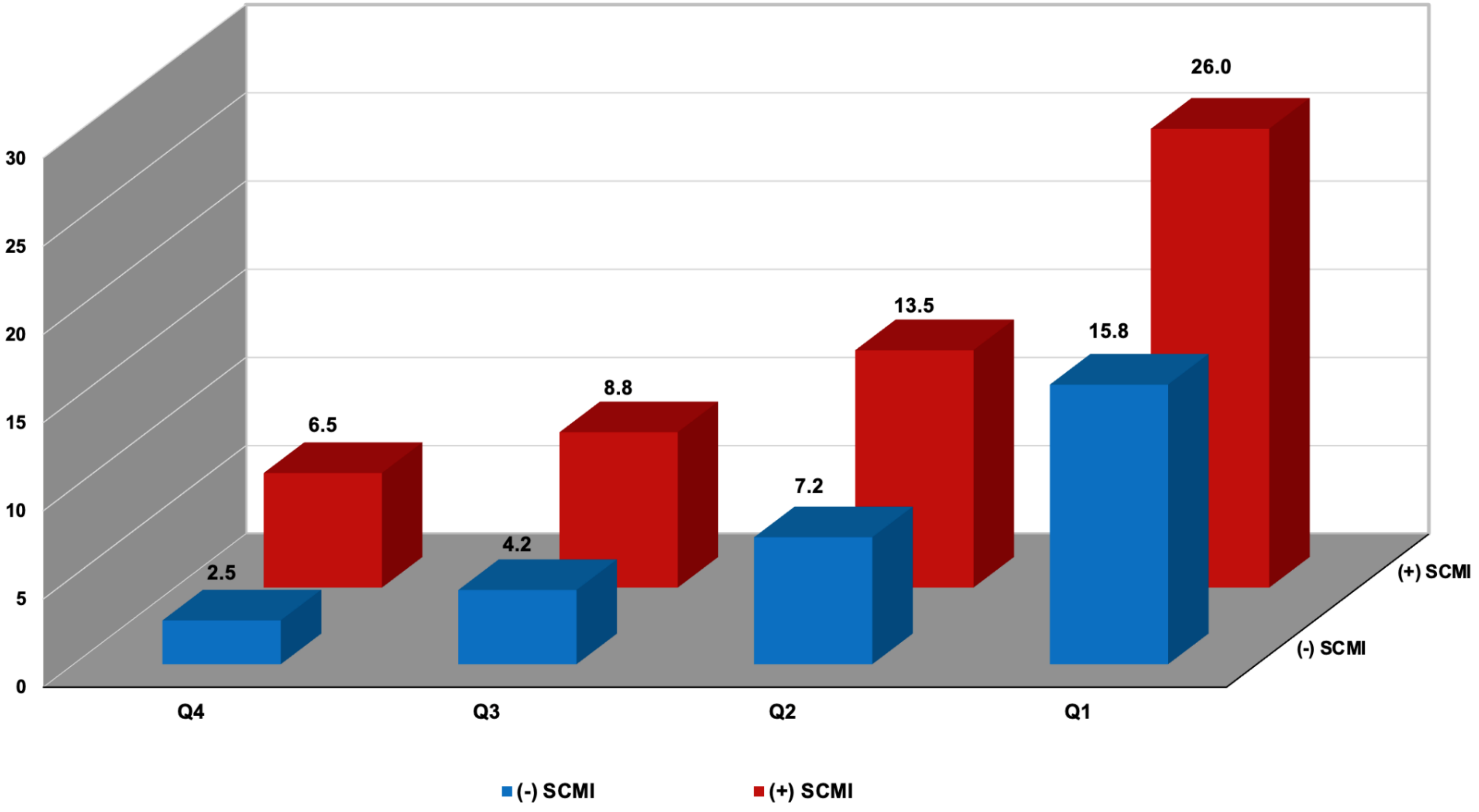

Incidence rate per 1,000 person-years
